# Persistent Left Superior Vena Cava in an Adult Presenting With Atrial Fibrillation

**DOI:** 10.7759/cureus.106614

**Published:** 2026-04-07

**Authors:** Chris Sani, Varshitha Panduranga, Francesca Cali, Asher Gorantla, Sabu John

**Affiliations:** 1 Internal Medicine, State University of New York Downstate Medical Center, Brooklyn, USA; 2 Cardiology, State University of New York Downstate Medical Center, Brooklyn, USA

**Keywords:** atrial arrhythmia, atrial fibrillation (af), bovine arch, bubble study, cardinal vein, congenital aortic arch anomaly, congenital cardiovascular malformation, persistent left superior vena cava (plsvc), transthoracic echocardiogram

## Abstract

Persistent left-sided superior vena cava (PLSVC) is usually an asymptomatic, rare congenital anomaly, but has been associated with atrial arrhythmias due to remnant pacemaker tissue within the vessel. This report describes a 62-year-old male who presented with new-onset atrial fibrillation (AF) and was incidentally found to have a PLSVC draining into a dilated coronary sinus, along with additional thoracic and vascular abnormalities, including a bovine aortic arch and tracheal diverticulum. Transthoracic echocardiography with agitated saline injection and computed tomography confirmed the diagnosis. The patient’s AF self-terminated and was managed medically, with referral for outpatient electrophysiology follow-up given the recognized association between PLSVC and supraventricular arrhythmias. This case highlights the importance of recognizing PLSVC during the evaluation of AF, the utility of multimodality imaging in establishing the diagnosis, and the need for further studies to clarify the arrhythmogenic potential of PLSVC and the role of targeted electrophysiologic intervention in adults.

## Introduction

A persistent left-sided superior vena cava (PLSVC) is a congenital vascular anomaly that occurs when the left anterior cardinal vein fails to regress during early embryonic development [1]. In normal development, the right anterior cardinal vein remains to become the superior vena cava. Although typically asymptomatic and without significant hemodynamic consequences, it has been associated with clinically significant complications, including arrhythmias [2]. One study found that PLSVC was present in approximately 0.9% of patients with atrial fibrillation (AF) [3]. Among those who underwent catheter ablation, PLSVC was identified as a trigger or driver of AF in 68.8% of patients [3]. The proposed arrhythmogenic mechanism involves residual muscular and pacemaker tissue within the PLSVC, which may serve as a nidus for the initiation of AF [4]. This case describes a patient presenting for new-onset AF with rapid ventricular response in which a previously undiagnosed PLSVC was identified during evaluation, emphasizing the importance of recognizing this anatomical variant as a potential contributor to arrhythmogenesis and its implications for management.

## Case presentation

**Figure 1:**
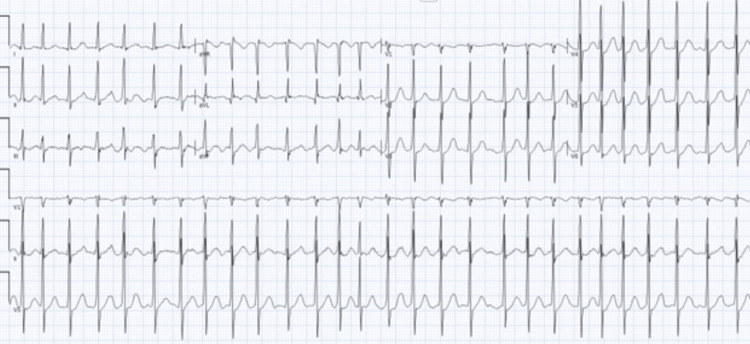
Electrocardiogram showing atrial fibrillation with a rapid ventricular rate of 168 beats per minute

A 62-year-old male presented to the emergency department with complaints of chest pain, palpitations, and dyspnea both at rest and with exertion. His past medical history included hypertension, hyperlipidemia, peptic ulcer disease, chronic back pain, and left anorchia. Vital signs were notable for a blood pressure of 122/61 mmHg and a heart rate of 87 beats per minute. An initial electrocardiogram (ECG) revealed AF with rapid ventricular response and ST-segment depression in the inferior and lateral leads (Figure 1). Initial laboratory studies revealed an elevated serum creatinine, high-sensitivity troponin levels that were negative, and a normal B-type natriuretic peptide level. The thyroid function tests and the remainder of the laboratory evaluation were within normal limits (Table 1). A chest radiograph was reported as showing prominent bilateral pulmonary arteries. While on telemetry in the emergency department, the AF spontaneously converted to sinus rhythm.

**Table 1:** Initial laboratory findings Repeat high-sensitivity troponin obtained three hours after the initial laboratory tests.

Laboratory Test	Result	Reference Range	Interpretation
White Blood Cell (WBC) Count	5.91 K/µL	4.5-10.9 K/µL	Normal
Hemoglobin	15.4 g/dL	14-18 g/dL	Normal
Sodium	143 mmol/L	136-146 mmol/L	Normal
Potassium	4.1 mmol/L	3.5-5.0 mmol/L	Normal
Chloride	106 mmol/L	98-106 mmol/L	Normal
CO₂ (Bicarbonate)	25 mmol/L	24-31 mmol/L	Normal
Blood Urea Nitrogen (BUN)	17 mg/dL	8-23 mg/dL	Normal
Creatinine	1.6 mg/dL	0.7-1.2 mg/dL	Elevated
Thyroid-Stimulating Hormone (TSH)	1.83 µIU/mL	0.27-4.2 µIU/mL	Normal
Free T4	1.2 ng/dL	0.93-1.7 ng/dL	Normal
High-Sensitivity Troponin (Initial)	34 ng/L	0-22 ng/L	Elevated
High-Sensitivity Troponin (Repeat)	28 ng/L	0-22 ng/L	Elevated
Pro-BNP	51 pg/mL	1-125 pg/mL	Normal

**Figure 2:**
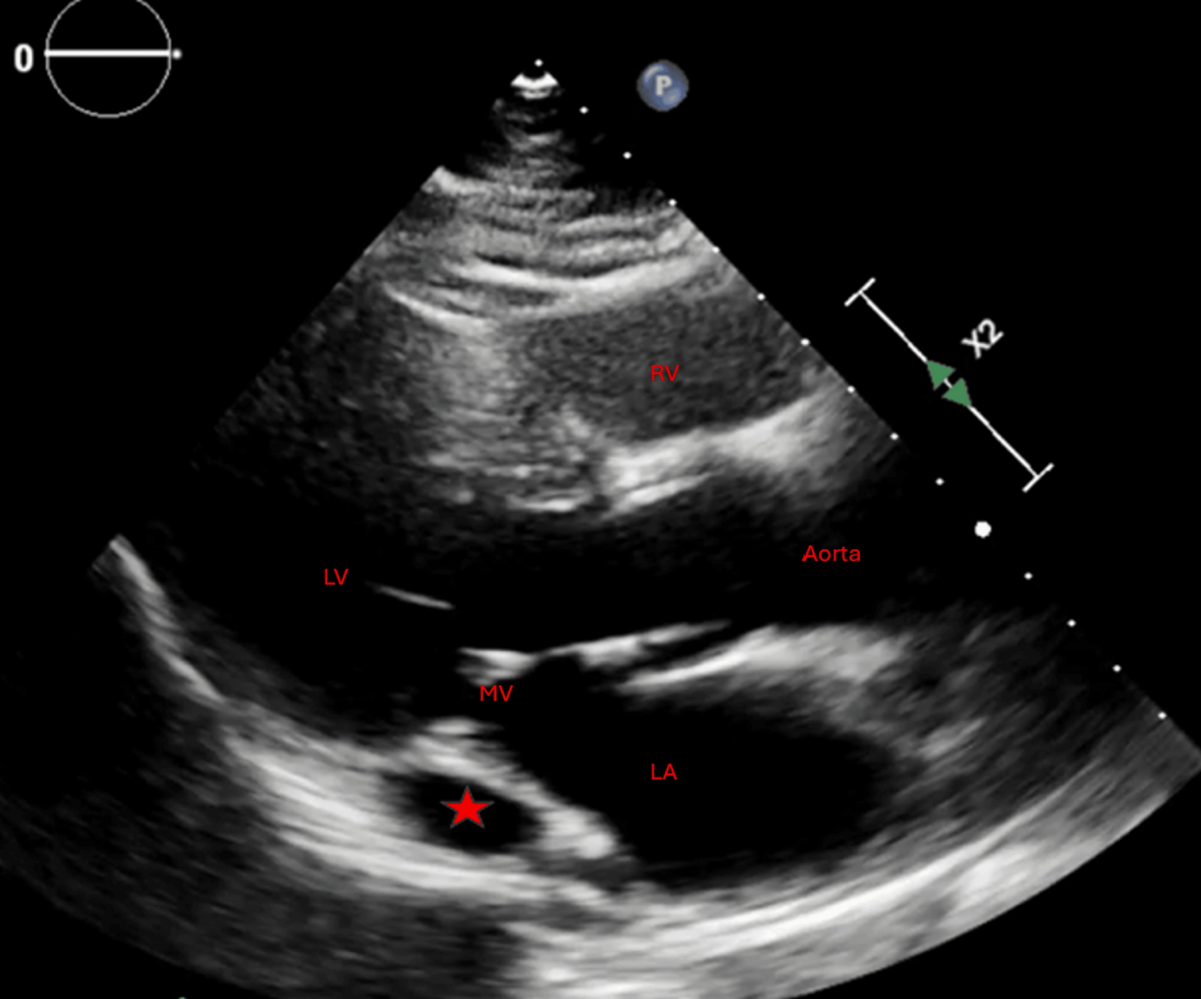
A transthoracic echocardiogram in parasternal long-axis view showing a dilated coronary sinus (red star) The parasternal long axis (PLAX) view is achieved by placing a phased array transducer in the third or fourth intercostal space just to the left of the sternum, with the probe marker directed toward the patient's right shoulder. LA: left atrium; LV: left ventricle; MV: mitral valve; RV: right ventricle

The patient was admitted for observation, and the cardiology service was consulted. CHA₂DS₂-VASc score of 1, which puts the patient at 0.6% risk of stroke. After the risks and benefits of anticoagulation were discussed with the patient, the patient was started on apixaban 5 mg every 12 hours based on combined decision-making. The patient was also started on rate control therapy with metoprolol tartrate 12.5 mg twice daily. A transthoracic echocardiogram was performed, which revealed a dilated coronary sinus in the parasternal long-axis view (PLAX) (Figure 2). A repeat limited study was performed to assess for PLSVC. Agitated saline was injected through an intravenous line in the left arm. In the PLAX view, the coronary sinus appeared dilated, and bubbles appeared in the coronary sinus before appearing in the right ventricle (Video 1).

**Video 1:** Transthoracic echocardiogram in parasternal long-axis view showing agitated saline initially draining into the coronary sinus (CS in red), followed by the right ventricle (RV in red)

**Figure 3:**
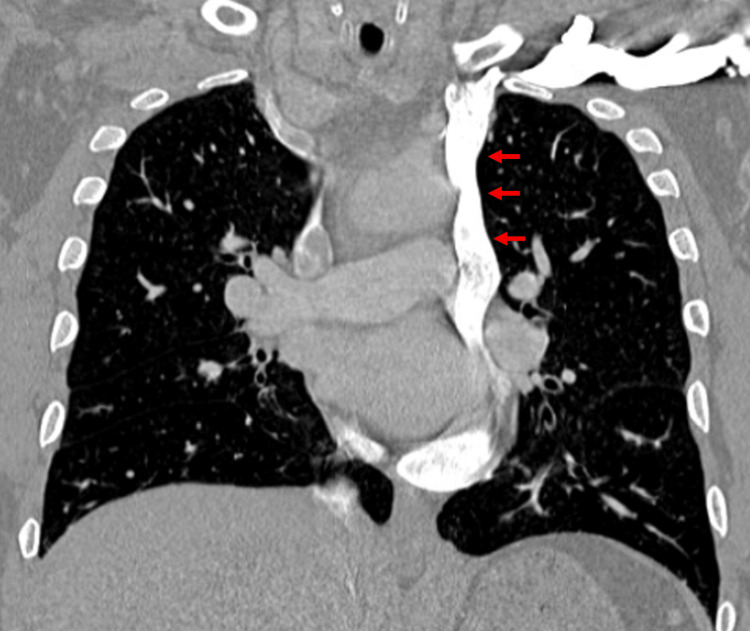
CT chest with contrast showing persistent left-sided superior vena cava (PLSVC) (red arrows) draining near the left heart or the coronary sinus

**Figure 4:**
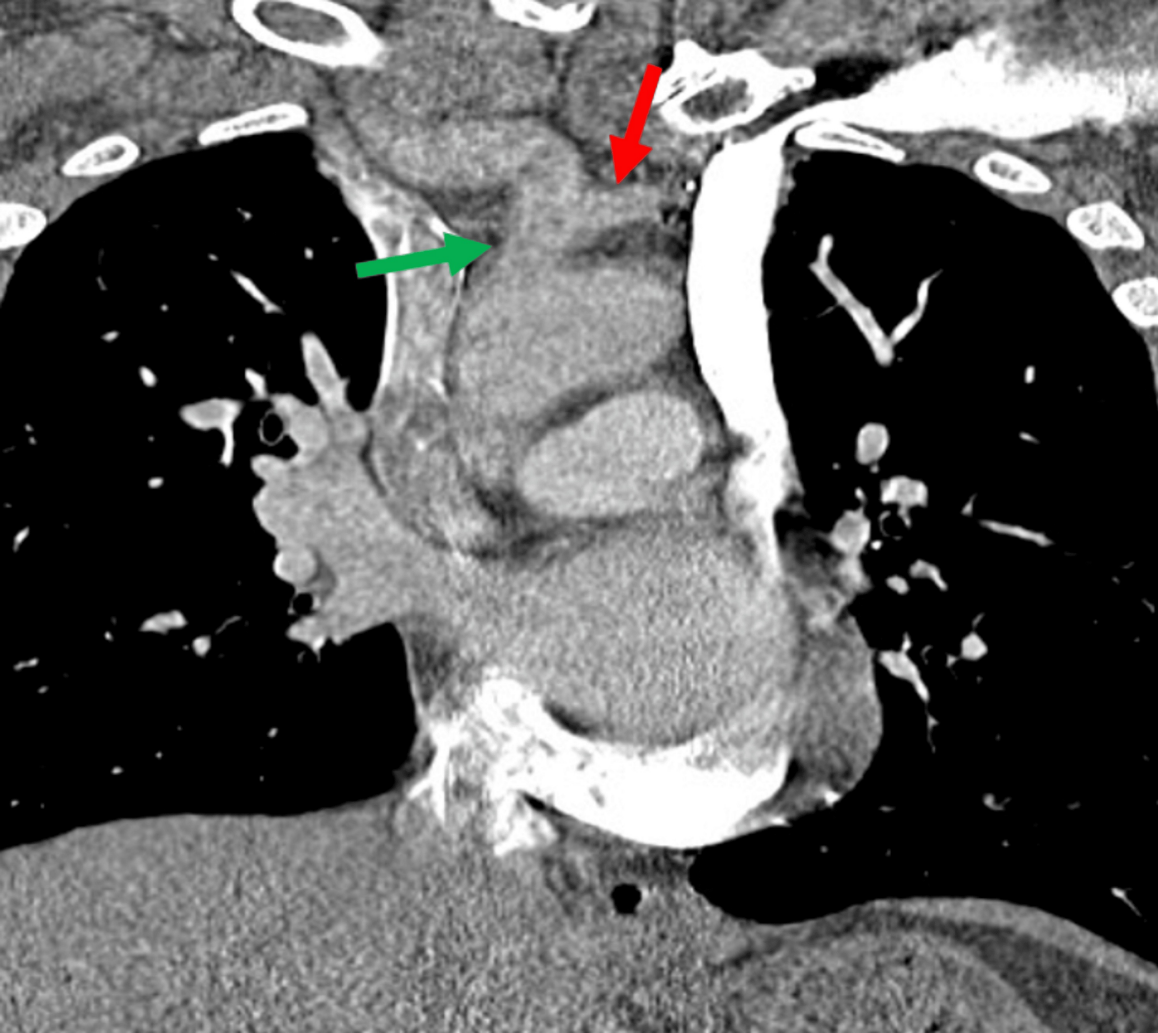
Left common carotid (red arrow) arising from the brachiocephalic trunk (green arrow)

A computed tomography (CT) scan of the chest with contrast was performed, which showed a PLSVC draining into the coronary sinus (Figure 3), the left common carotid artery arising from the brachiocephalic trunk (Figure 4), a nonspecific focus of air to the right of the esophagus and trachea likely representing a tracheal diverticulum (less likely an esophageal diverticulum), a small hiatal hernia, and diffuse cylindrical bronchiectasis. The diagnosis of PLSVC was made. General surgery was consulted, and outpatient follow-up was recommended given the non-emergent nature of the finding. A stress ECG and echocardiogram were reported as sinus tachycardia without ST segment changes, arrhythmias, or post-stress wall motion abnormalities. At discharge, his metoprolol tartrate dosage was increased to 50 mg twice daily for improved rate control, and he continued apixaban 5 mg twice daily. The patient was counseled regarding the incidental finding and was referred for outpatient electrophysiology follow-up due to his increased risk of arrhythmia with PLSVC. He was instructed to report his anatomical findings during future hospitalizations, particularly if central venous access or ablation procedures are required. He was also advised to inform his primary care provider to ensure appropriate follow-up and further evaluation of the thoracic abnormalities.

## Discussion

A PLSVC is a rare congenital vascular abnormality that arises within the thorax. In embryonic development, it results from the persistence of the left anterior cardinal vein, which normally regresses to form the ligament of Marshall. PLSVC is present in approximately 4.5% of patients with congenital heart disease, particularly those with atrial septal defects [4]. This patient demonstrated multiple thoracic abnormalities, including a probable tracheal diverticulum and a left common carotid artery arising from the brachiocephalic trunk. In 80-90% of cases, PLSVC drains into the coronary sinus, whereas in 10-20% of cases, it drains into the left atrium [5]. Aortic branch abnormalities are associated with PLSVC, including a right-sided aortic arch and coarctation of the aorta; however, a left common carotid artery arising from the brachiocephalic trunk (often referred to as a “bovine arch”) has only rarely been described in association with PLSVC and has previously been reported primarily in a fetal case [6].

While most patients with PLSVC are asymptomatic, the anomaly has been associated with an increased risk of arrhythmias, as well as symptoms such as decreased exercise tolerance, palpitations, syncope, and cyanosis. Embryologically, the early heart contains two symmetrically located pacemaker regions. The right atrial pacemaker ultimately assumes function and becomes the sinoatrial node; however, the embryonic left-sided pacemaker tissue migrates to its final location near the coronary sinus [4]. Because of these remnants, PLSVC may serve as a potential arrhythmogenic substrate, although AF appears to be less common than other supraventricular tachycardias (SVTs). In a study by Hwang et al., among 6,662 patients who underwent electrophysiologic studies for SVT, 18 were found to have PLSVC. Of these 18 patients, seven had atrioventricular nodal reentrant tachycardia (AVNRT), four had accessory pathway-mediated tachycardia, four had atrial tachycardia, and only two had AF [7].

It is hypothesized that since PLSVC maintains electrical connections to the left atrium and coronary sinus, it can serve as a conduit for ectopic activity, including AF. Electroanatomic mapping is therefore essential for identifying arrhythmogenic foci within the PLSVC, and patients should undergo cardiac CT or MRI prior to electrophysiologic studies [3]. Selective isolation of the PLSVC, in addition to pulmonary vein isolation, has been associated with favorable outcomes in PLSVC-triggered supraventricular tachyarrhythmias, including AF [8,9]. However, ablation within the PLSVC is not without risks, as procedures involving the PLSVC have been associated with complications such as phrenic nerve injury and cardiac tamponade [3,9]. Thus, careful procedural planning is essential, and interventions involving PLSVC should be performed at tertiary centers with cardiothoracic surgical expertise available to manage potential complications.

## Conclusions

While PLSVC is a common thoracic venous anomaly and is frequently associated with congenital heart disease and aortic abnormalities, the combined presence of PLSVC and a left common carotid artery arising from the brachiocephalic trunk appears to be rarely reported in the adult literature. Although PLSVC has been described to possess intrinsic pacemaker activity and has been associated with supraventricular tachyarrhythmias, its relationship with AF remains less well defined. This case highlights the importance of recognizing PLSVC as a potential contributor to arrhythmogenesis and underscores the need for heightened awareness of associated vascular variants that may have implications for diagnostic evaluation and procedural planning. Further studies are warranted to better characterize the electrophysiologic properties of PLSVC and to clarify the risks and long-term outcomes associated with PLSVC-directed ablation in adults.
